# Validation and assessment of preanalytical factors of a fluorometric in vitro assay for glucocerebrosidase activity in human cerebrospinal fluid

**DOI:** 10.1038/s41598-020-79104-5

**Published:** 2020-12-16

**Authors:** Linn Oftedal, Jodi Maple-Grødem, Marthe Gurine Gunnarsdatter Førland, Guido Alves, Johannes Lange

**Affiliations:** 1 The Norwegian Centre for Movement Disorders, Stavanger University Hospital, Stavanger, Norway; 2 Department of Psychiatry, Centre for Age-Related Medicine, Stavanger University Hospital, Stavanger, Norway; 3 Department of Chemistry, Bioscience and Environmental Engineering, University of Stavanger, Stavanger, Norway; 4 Centre for Organelle Research, University of Stavanger, Stavanger, Norway; 5 Department of Neurology, Stavanger University Hospital, Stavanger, Norway

**Keywords:** Analytical biochemistry, Biomarkers, Neurological disorders

## Abstract

Lysosomal dysfunction is an emerging feature in the pathology of Parkinson’s disease and Dementia with Lewy bodies. Mutations in the *GBA* gene, encoding the enzyme Glucocerebrosidase (GCase), have been identified as a genetic risk factor for these synucleinopathies. As a result, there has been a growing interest in the involvement of GCase in these diseases. This GCase activity assay is based on the catalytic hydrolysis of 4-methylumbelliferyl β-d-glucopyranoside that releases the highly fluorescent 4-methylumbelliferyl (4-MU). The final assay protocol was tested for the following parameters: Lower limit of quantification (LLOQ), precision, parallelism, linearity, spike recovery, number of freeze–thaw events, and sample handling stability. The GCase activity assay is within acceptable criteria for parallelism, precision and spike recovery. The LLOQ of this assay corresponds to an enzymatic activity of generating 0.26 pmol 4-MU/min/ml. The enzymatic activity was stable when samples were processed and frozen at − 80 °C within 4 h after the lumbar puncture procedure. Repetitive freeze–thaw events significantly decreased enzyme activity. We present the validation of an optimized in vitro GCase activity assay, based on commercially available components, to quantify its enzymatic activity in human cerebrospinal fluid and the assessment of preanalytical factors.

## Introduction

Evidence points towards impairment of lysosomal mechanisms as a key event in the susceptibility and pathogenesis of the Parkinson’s disease (PD) and Dementia with Lewy bodies (DLB)^1^. The most commonly known genetic risk factor for development of these synucleinopathies are mutations in the *GBA* gene^2^. This gene encodes the enzyme glucocerebrosidase (GCase) that catalyzes the hydrolytic cleavage of glycosphingolipids^3^. Decreased GCase activity leads to an accumulation of glycosphingolipids followed by lysosomal dysfunction and stabilization of toxic α-synuclein species^4^.

Patients with PD and DLB exhibit decreased GCase enzymatic activity in affected brain regions^2^. Moreover, reduced GCase activity has been found in substantia nigra of PD and DLB patients independent of their *GBA* mutation status^5^. The identification of impaired GCase activity in PD and DLB pathologies has provided a link between these neurological diseases and lysosomal dysfunction^3^. Defective function of GCase is in fact the cause of Gaucher disease (GD), the most common lysosomal storage disorder. The definite diagnosis of GD is given by the beta-glucosidase leukocyte assay using the same substrate^6,7^, 4-methylumbelliferyl β-d-glucopyranoside, that is used to assess GCase activity in PD and DLB.

A few studies, originating from one group, have evaluated GCase activity in cerebrospinal fluid (CSF) of PD and DLB patients. Three studies have found significantly reduced GCase activity in PD patients^8–10^, while one study did not^11^. The only study addressing this in DLB found a reduction of GCase activity in CSF of DLB patients compared to controls^12^. As seen in brain samples, deficient GCase activity in CSF of PD patients cannot only be found in *GBA* mutation carriers, but also in non-carriers^10^. The notion that CSF β-glucocerebrosidase activity is reduced in PD patients independent of their *GBA* mutation carrier status suggests that GCase activity could be a valuable biomarker for idiopathic forms of PD and DLB and as an objective outcome measure in clinical trials^13^, and further studies are warranted.

In this study, we have optimized a fluorometric assay to quantify GCase activity in CSF to reduce sample consumption and improve sensitivity. To determine its reliability and reproducibility we validated the assay by measuring precision, parallelism, linearity and spike-recovery. In addition, we assessed important preanalytical conditions, including number of freeze–thaw events and sample handling stability, and determined lower limit of quantification (LLOQ).

## Materials and methods

### GCase activity assay

#### Assay and sample preparation

CSF samples were thawed on ice, centrifuged briefly at 1000×*g* and diluted 1:2 in assay buffer (0.1 M citric acid (#84841.290, VWR Chemical, USA) and 0.2 M Na_2_HPO_4_ (#28026.260, VWR Chemicals, USA), pH 5, supplemented with 2 mg/ml taurodeoxycholic acid (TDC, #336840010, Acros organics, Belgium)) prior to the assay. The GCase substrate, 4-methylumbelliferyl β-d-glucopyranoside (#J66630.MD, Sigma Aldrich, USA), was dissolved at a concentration of 0.5 mM in assay buffer. 4-methylumbelliferyl (4-MU, #A10337, Alfa Aesar, USA) served as a calibrator.

#### Assay procedure

We modified and optimized the assay described by van Dijk et al.^11^ to decrease sample consumption and increase sensitivity. The wells of a black 96-well plate (#3991, Corning, USA) were filled with 15 µl diluted samples and wells reserved for calibrators or blanks were filled with 15 µl assay buffer. Then, 30 µl substrate solution were added to each well and plates were sealed (#732–4838, VWR International, USA). After shaking for 3 min at 600 rpm, the plates were incubated at 37 °C for 3 h. Within the last hour of incubation, a twofold serial dilution of 4-MU in stop solution (0.2 M glycine (#36,435.30, Alfa Aesar, USA)-NaOH (#1.06498.1000, Merck KGaA, Germany), pH 10.4) giving a final concentration in the wells of 100, 50, 25, 12.5, 6.25, 3.13 and 1.56 nM was prepared. At the end of the incubation period, 180 µl stop solution was added to each well containing sample and 180 µl of the calibrator dilutions were added into the wells reserved for calibrator. Six wells were reserved for the blank (containing assay buffer with GCase substrate and added stop buffer). The plates were shaken briefly to ensure complete mixing and then read within 30 min on a Synergy H1m multimode reader (Biotek, USA) in fluorescence mode with excitation at 360 nm and emission at 446 nm. All samples and calibrators were run in triplicates. All buffers were filtered through a 0.2 µm filter (#28415-483, VWR International, USA) and plates were washed with MilliQ-water (Merck KGaA, Germany) before use. A user guide is included in the supplementary material online.

### CSF samples

For method development and validation, anonymized leftover samples from clinical routine were obtained at the department of Neurology at Stavanger University Hospital, Norway, by qualified health professionals following standard procedures and ethics guidelines granted by the Regional Committee for Medical and Health Research Ethics of Western Norway (REC West, issued June 11th, 2012). All CSF donors provided informed consent to lumbar puncture as part of their diagnostic workup. In addition, for measurements of GCase activity in PD, a set of 19 samples was available. Donors signed written informed consent and all procedures and ethical guidelines were approved by REC. All PD samples had hemoglobin concentrations below 200 ng/ml (#E88-134, Bethyl Laboratories, USA).

After lumbar puncture (LP), all CSF samples were immediately placed on ice, centrifuged at 2000×*g* for 10 min at 4 °C. Samples were aliquoted into portions of same volume and frozen by placing them on dry ice prior to long term storage at − 80 °C. Storage time for CSF samples was between 2 weeks and 5 years. Samples were subjected to one freeze–thaw event for aliquotation purposes prior to analysis. For method development, samples with visible blood contamination were excluded. Persichetti et al.^14^ showed that a blood contamination of up to 50,000 erythrocytes per microliter CSF did not significantly impact GCase activity measurements. This study complies with The Code of Ethics of the World Medical Association (Declaration of Helsinki).

### Validation of detection method

#### Design

Assay validation methods were selected to test critical assay parameters listed below, using definitions adapted from published guidelines for immunoassays^15^. Both pooled and individual samples were used in the validation experiments and sample numbering is consistent throughout.

#### Lower limit of quantification (LLOQ)

To determine the LLOQ, the signal of sixteen blank replicates (containing assay buffer with GCase substrate and stop buffer) was measured (background). The LLOQ was calculated as the concentration of 4-MU corresponding to the mean signal of the sixteen replicates plus ten times the standard deviation (SD). The upper limit of quantification is dependent on the fluorescence reader instrument. On the H1m reader, 4-MU concentrations over 125 nM resulted in detector saturation with standard gain settings.

#### Parallelism

Five CSF samples were diluted 1:2 in assay buffer and then further diluted in twofold steps until a final dilution of 1:16. All dilutions and undiluted samples were subjected to the assay protocol and analyzed in triplicate. Parallelism assesses the similarity between the dose–response curves of the calibrator and the enzymatically-generated 4MU in the samples.

#### Assessments of linearity

(1) Five CSF samples were analyzed in quadruplicate with the incubation step at 37 °C being stopped after 2, 3, 4 or 5 h respectively (triplicate wells for each time point). Linearity of the GCase activity over time was assessed by linear regression of incubation time versus 4-MU concentration. (2) Four CSF samples were spiked with six different concentrations of recombinant GCase (#7410-GHB, R&D Systems, USA) ranging from 5 to 160 pM. To mimic very low endogenous GCase levels, another four CSF samples were subjected to thermal denaturation by heating to 95 °C for 5 min in a dry bath incubator and thereafter spiked with recombinant GCase as described above. Endogenous GCase activity was subtracted from measured 4-MU concentration and the linearity of the dose–response relationship assessed by linear regression.

#### Spike-recovery

Five CSF samples were diluted in assay buffer (1:2) and spiked with 4-MU to a final concentration of either 3.125 (approximately two times LLOQ), 6.25 or 12.5 nM before being subjected to the standard assay protocol. Endogenous GCase activity was determined by analyzing non-spiked samples. 4-MU generated by endogenous GCase activity was subtracted from 4-MU concentration in spiked samples before calculation of recovery.

#### Precision

Five CSF samples were divided into small volume polypropylene tubes and frozen at − 80 °C. On five different occasions, a set of five aliquots for each CSF sample was analyzed, in total 25. Precision replicates were thawed independently and diluted independently on the day of the assay day. Each individual aliquot was run as in triplicate on the assay plate.

#### Preanalytical condition: time delay before freezing

For sample stability experiments, freshly drawn CSF samples were aliquoted and frozen in batches: the first batch was frozen within 1 h, the second batch after 2 h, the third batch after 4 h and the last batch after 24 h. Samples with delayed freezing were kept at 4 °C until being frozen. All four batches of five CSF samples were analyzed using the final assay protocol.

#### Preanalytical condition: freeze–thaw cycles

For freeze–thaw susceptibility experiments, pre-aliquotted samples were subjected to repeated freeze–thaw events ranging from one to five. Samples tubes were allowed to sit at − 80 °C for at least one week before subjecting the sample tube to an additional thawing and refreezing. Samples were completely thawed on ice and mixed by vortexing. Aliquots of four samples were included for this test and analyzed using the final assay protocol.

### Data analysis

Standards and samples were analyzed in triplicates. Standard curve fitting (linear regression), plotting of calibrator curve and calculation of concentrations were performed using the Gen5 Data Analysis software (Biotek, USA). All other calculations were performed in Excel (Microsoft, USA). For parallelism, absolute values were normalized to values of the assay-recommended dilution for better comparability. Acceptable limits for parallelism and spike recovery were 80–120%. Coefficients of variation (CV) < 20% for repetitive measurements and < 15% for intra-assay measurements were considered acceptable. Intra- and inter-assay CVs for precision samples were calculated according to ISO 5725-2 using the Excel sheet provided by Andreasson et al.^15^ as a supplementary file. Differences within preanalytical conditions (Number of repeated freeze–thaw events and Time from sampling to freezing) were assessed by Wilcoxon Signed Rank test in SPSS version 26 (IBM, USA). A *p* value of < 0.05 was considered statistically significant.

One unit (U) of GCase activity was defined as amount of enzyme that hydrolyses 1 nmol of substrate/min at 37 °C.

On rare occasions (~ 2%), distinctly higher fluorescence was read for single wells compared to their corresponding replicates. This could be minimized by filtration of buffers and washing of plates. To account for this, values were classified as outliers when they deviated at least two times from the mean of the other replicates. Outliers were omitted from calculations.

## Results

### Validation of the assay for the detection of GCase activity in human CSF

We modified and optimized the assay described by van Dijk et al.^11^ to determine GCase activity in CSF of PD patients. A reduction of the substrate concentration from 3 mM (van Dijk et al.^11^) to 0.5 mM improved the LLOQ by a factor of over 6.5 (3 mM: 1.84 pmol 4-MU/min/ml; and 0.5 mM: 0.26 pmol 4-MU/min/ml). We reduced sample consumption from 20 µl to 7.5 µl per well. The assay was then validated for the following characteristics:

#### Lower limit of quantification (LLOQ)

The lowest concentration of generated 4-MU that reliably can be quantified by this assay is 1.559 nM after 180 min. This corresponds to a LLOQ of 0.26 pmol 4-MU/min/ml or 0.26 mU/ml (1:2 dilution, 7.5 µl CSF per reaction). The mean signal of sixteen replicates of calibrator diluent was 2419.1 ± 103.6 signal units (CV = 4.3%), corresponding to 1.559 nM 4-MU, and considerably below typical sample values.

#### Parallelism

The assay showed good overall parallelism across undiluted to 1:8 dilution (mean recovery of all five samples 86%, CV% 11.6 against the assay dilution (1:2) we here determined to be the best, Table 1 and Supplementary Table S1 online). However, compared one by one, three undiluted samples and samples diluted 1:8 had a recovery below accepted criteria (80–120% recovery). A dilution of 1:16 resulted in a mean recovery of only 63%.

**Table 1:** Parallelism of five different CSF samples.

CSF	mU/ml at 1:2 dil	Recovery, %					Mean recovery (A, C & D) against assay dilution (B), %		
		A	B	C	D	E			
**Dilution**		**Neat**	**1:2**	**1:4**	**1:8**	**1:16**	**Mean**	**SD**	**CV%**
CSF 10	0.687	74	100	96	78	61	83	11.9	14.3
CSF 11	0.999	73	100	81	69	56	74	6.0	8.0
CSF 12	1.357	74	100	80	61	35	72	9.9	13.9
CSF 16	0.439	112	100	106	107	74	108	3.2	2.9
CSF 17	1.263	113	100	86	80	89	93	17.6	19.0
**Mean**		**89**	**100**	**90**	**79**	**63**	**86**	**9.7**	**11.6**
SD		21.3	0	11.1	17.3	20.3			
CV%		23.9	0	12.4	21.9	32.2			

#### Assessments of linearity

**Figure 1:**
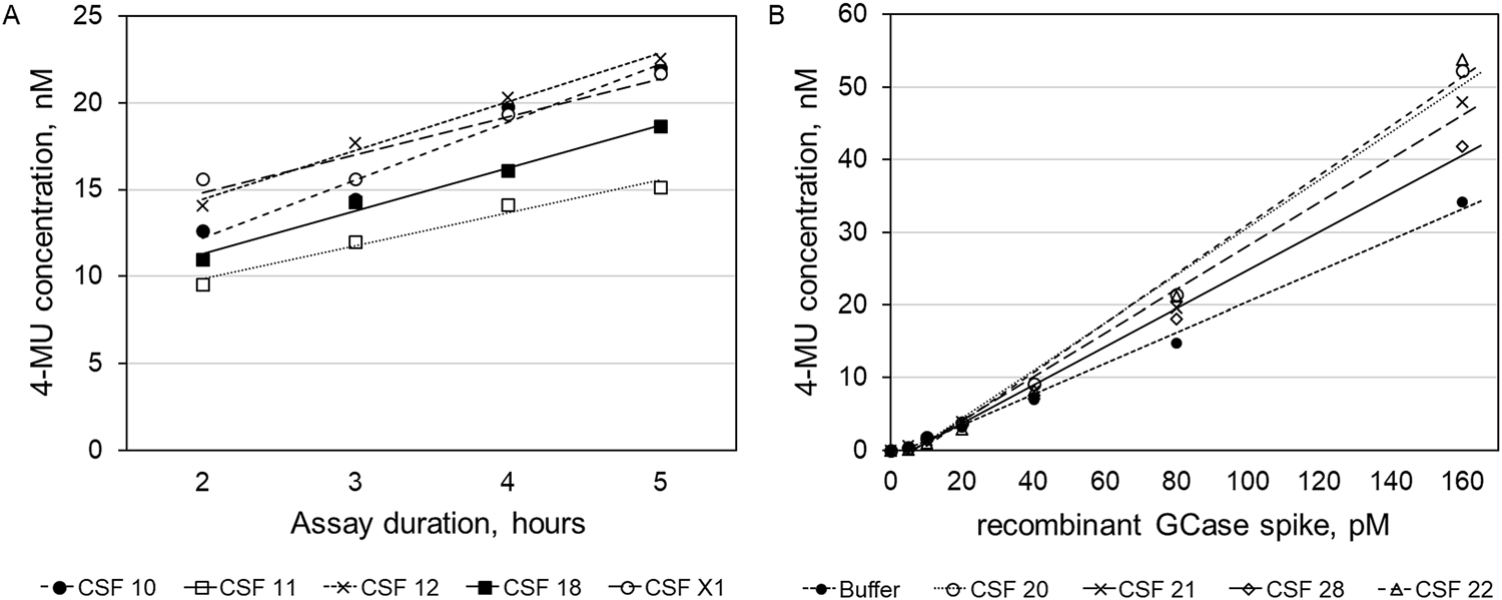
Linearity of the assay. (**A**) Samples were analyzed with this assay, varying the incubation step at 37 °C from 2 to 5 h. Linear regression R^2^ varied from 0.899 to 0.988. (**B**) Samples were spiked with recombinant GCase ranging from 5 to 160 pM. Linear Regression R^2^ varied from 0.986 to 0.995.

This assay uses a 3-h incubation step at 37 °C. The GCase activity response was linear for incubation step lengths of between 2 and 5 h (R^2^ > 0.899, Fig. 1A). The GCase activity measured was linear to the concentration of GCase spiked into the CSF sample matrix (R^2^ > 0.986, Fig. 1B) in the range between 5 and 160 pM. Similar results (R^2^ > 0.989) were found when the assay was repeated using thermodynamically denatured CSF, which exhibits lower endogenous levels of GCase activity.

#### Spike recovery: investigation of concentration–response relationship

Five CSF samples were spiked with three different concentrations, ranging from one to four times LLOQ, of 4-MU (Table 2). Mean spike recovery was between 96 and 102% and recovery was within acceptable limits (80–120%) for all CSF samples at all spike concentrations.

**Table 2:** Spike recovery of five different CSF samples.

Spike, nM			
	3.125	6.25	12.5
**Recovery, %**			
CSF 10	92	97	100
CSF 11	90	99	99
CSF 12	93	85	95
CSF 16	103	104	108
CSF 17	104	104	110
**Mean**	**96**	**98**	**102**
SD	6.6	8.1	6.5
CV	6.9	8.3	6.4

#### Precision: determination of intra-assay and inter-assay variability

Intra-assay CV%s (repeatability) were between 2.6 and 13.7 for the five CSF samples (Table 3). Inter-assay CV%s (intermediate precision) were between 3.3 and 14.9. Both intra-assay variation and inter-assay variation were within acceptable limits.

**Table 3:** Precision of five different CSF samples.

		Repeatability	Intermediate precision
Sample ID	Mean value (mU/ml)	%CV_r_	%CV_Rw_
CSF 7	0.867	6.6	7.9
CSF 8	0.942	8.9	9.0
CSF 13	0.483	13.6	14.9
CSF 14	1.083	2.6	3.3
CSF 15	0.850	7.3	8.3
**Mean**		**7.8**	**8.7**
Range	0.483–1.083	2.6–13.7	3.3–14.9

#### Preanalytical condition: time delay before freezing

**Figure 2:**
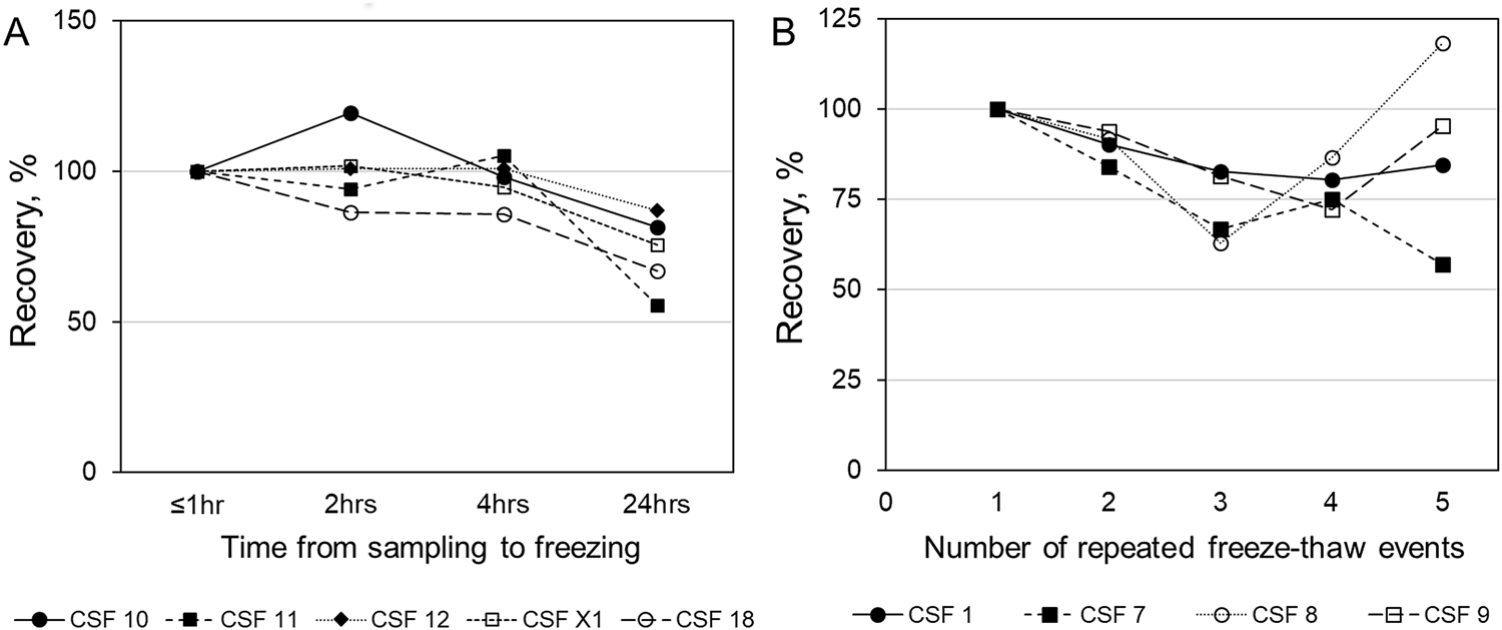
Preanalytical conditions affecting GCase activity in CSF samples. (**A**) Stability of the GCase activity in CSF upon time-delay before freezing. After LP, samples were aliquoted and kept at 4 °C before being frozen within 1 h, 2 h, 4 h or 24 h. (**B**) Stability of the GCase activity assessed in four CSF samples after freeze–thaw events.

Several of the most common pre-storage conditions, that is storage of CSF for up to 4 h after LP at 4 °C before freezing, were tested to assess the stability of GCase activity upon such sample handling. As an extreme, we included samples stored at 4 °C for 24 h after LP as well. The GCase activity was stable in samples that were processed to freezing within 4 h after sampling. An average reduction of 27% in GCase activity could be observed in samples that were frozen 24 h after sampling (Fig. 2A, *p* < 0.05 vs after 1 h and after 2 h, respectively).

#### Preanalytical condition: freeze–thaw events

Repeated thawing and refreezing affected the GCase activity (Fig. 2B, differences statistically not significant). After five freeze–thaw events the mean GCase activity of four different samples had decreased to 88.9% with a CV% of 28.6. However, the effect of the freeze–thaw cycle on the GCase activity was variable across samples and was more coinciding with less freeze–thaw events.

#### GCase levels in PD samples

**Figure 3:**
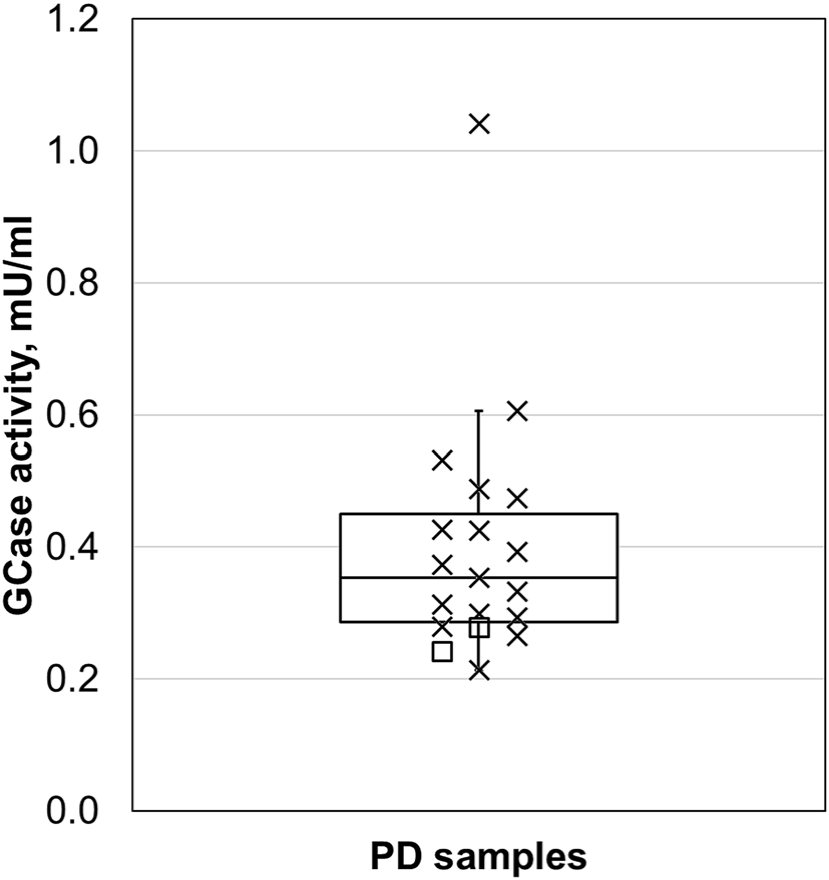
GCase levels in 19 individuals with idiopathic PD. GCase activity ranged from 0.22 to 1.04 mU/ml with CV% values < 9.4. Individual GCase activities of GBA polymorphism carriers are indicated as squares, non-GBA polymorphism carriers as ‘x’.

This validated assay is suitable to analyze GCase activity in PD samples. All 19 PD samples, including two GBA polymorphism carriers, were within the detection range with CV%-values below 9.4. (Fig. 3).

## Discussion

The potential of GCase as a biomarker in neurodegenerative diseases, especially PD, deserves attention and needs to be explored. To determine its suitability as a biomarker candidate, validated and accessible detection techniques are essential. We aimed to optimize and validate a GCase activity assay for CSF samples^12^, which has been implemented by an Italian group for PD and DLB CSF samples^8–12,14^. All materials are commercially available and the assay is easy to implement and cost-efficient. Optimization included use of less substrate (0.5 mM versus 3 or 10 mM) and sample consumption (7.5 µl vs. 20 µl). The chosen design of validation experiments included critical assay parameters like LLOQ, precision, parallelism, linearity and spike recovery.

Our *in vitr*o GCase activity assay is the first to determine the important parameters parallelism and spike recovery in CSF samples. Even though fluorometric GCase activity assays have been used previously to determine GCase activity in CSF samples, systematic analysis of preanalytical parameters have only been addressed once by Persichetti et al. ^14^, who concluded that GCase activity is stable for 32 weeks at − 80 °C, when kept cold after LP and frozen within short time. We examined the effect of common preanalytical conditions by investigating time delay before freezing and freeze–thaw events. Our results were in accordance with previous findings for stability of GCase activity in CSF^14^. We conclude that CSF samples should be kept cooled and either processed or frozen within 4 h after LP. Based on the results, we also recommend that repeated thawing and refreezing should be avoided and for comparisons, the samples should be subjected to the same number of freeze–thaw events. We further showed that the dose–response relationship is linear over the incubation time in a range of 2–5 h and linear to the concentration of GCase present in the sample. This study underscores the importance of assessment of preanalytical factors in accordance to obtain reliable GCase activity results.

Our assay passed the validation criteria for precision, parallelism and spike recovery showing that our in vitro GCase activity assay is reliable and reproducible when preanalytical factors like sample processing and freeze–thaw events are taken care of. Because GCase activity is reported to be lower in PD^8–10^ and to show the suitability of the assay, we analyzed 19 PD samples including two GBA polymorphism carrier. All samples were within the detection range of the assay and had a CV% below 10. The very lowest activities were slightly below the previously determined LLOQ but still within detection range. The assay also has the potential for further development to an assay to screen for inhibitors and activators of GCase^16^ and be used to monitor treatment targeting GCase^13^. Given the excellent assay performance, we will employ this assay with clinical cohorts to further determine the possibility of using GCase activity as a diagnostic and prognostic biomarker for PD and DLB.

## Conclusion

By optimising the GCase activity assay in cerebrospinal fluid, we were able to develop a reliable method that uses lower sample volumes and lower substrate concentration whilst also delivering increased sensitivity. To obtain reliable results, sample handling and freeze–thaw events must be considered, since these preanalytical factors were shown to affect GCase activity.

## Supplementary information

Supplementary Information.

## Data Availability

Data is available upon request.

## Abbreviations

CSF: Cerebrospinal fluid
GCase: Glucocerebrosidase
4-MU: 4-Methylumbelliferyl
LLOQ: Lower limit of quantification
PD: Parkinson’s disease
DLB: Dementia with Lewy bodies
GD: Gaucher disease
LP: Lumbar puncture
