# Supplementary material for: Validation and assessment of preanalytical factors of a fluorometric in vitro assay for glucocerebrosidase activity in human cerebrospinal fluid
Source: Sci Rep. 2020 Dec 16;10:22098. doi: 10.1038/s41598-020-79104-5 (PMC7744549; doi:10.1038/s41598-020-79104-5)
Supplement: Supplementary file 1 — Supplementary Information. [file 41598_2020_79104_MOESM1_ESM.docx]

**Validation and assessment of preanalytical factors of a fluorometric in vitro assay for glucocerebrosidase activity in human cerebrospinal fluid**

Linn Oftedal^1,2^, Jodi Maple-Grødem^1,3^, Marthe Gurine Gunnarsdatter Førland^1,4^, Guido Alves^1,3,5^, Johannes Lange^1,4,^*

^1^ The Norwegian Centre for Movement Disorders, Stavanger University Hospital, Stavanger, Norway

^2^ Centre for Age-related Medicine, Department of Psychiatry, Stavanger University Hospital, Stavanger, Norway

^3^ Department of Chemistry, Bioscience and Environmental Engineering, University of Stavanger, Stavanger, Norway

^4^ Centre for Organelle Research, University of Stavanger, Stavanger, Norway

^5^ Department of Neurology, Stavanger University Hospital, Stavanger, Norway

*** Corresponding author**: **Dr. Johannes Lange**, The Norwegian Centre for Movement Disorders, Stavanger University Hospital, Postboks 8100, Stavanger 4068, Norway; and Centre for Organelle Research, University of Stavanger, Stavanger, Norway, Phone: +47-5151-56-02, E-mail: [Johannes.lange@sus.no](mailto:Johannes.lange@sus.no)

**Supplementary material online**

**Table S1. Stepwise parallelism of five different CSF samples.**

|  | | **Mean recovery, % (CV%)** | | | | |
| --- | --- | --- | --- | --- | --- | --- |
|  | | **to dilution** | | | | |
|  | | **neat** | **1:2** | **1:4** | **1:8** | **1:16** |
| **from dilution** | **neat** | - | 117.1 (22.4) | 104.0 (19.6) | 89.6 (14.9) | 70.3 (20.5) |
|  | **1:2** | 89.0 (23.9) | - | 89.8 (12.4) | 78.9 (21.9) | 63.0 (32.2) |
|  | **1:4** | 99.4 (21.1) | 112.6 (11.7) | - | 87.2 (11.2) | 70.0 (31.2) |
|  | **1:8** | 113.8 (16.0) | 131.3 (19.8) | 115.8 (11.1) | - | 79.6 (25.5) |
|  | **1:16** | 148.3 (25.0) | 175.0 (38.2) | 154.2 (31.2) | 131.7 (23.4) | - |

**User guide**

STOCK SOLUTIONS AND BUFFERS

4MU stock solution [1mM] Dissolve 4MU (#A10337, Alfa Aesar, USA) completely in DMSO to make a 100mM solution. Dilute to 1mM by adding 0.1mL of this 100mM solution to 9.9mL DMSO.

4MU working solution [10µM] Dilute 4MU stock (1mM) solution 1:100 with MilliQ water.

4MU calibrator solution Dilute 4MU working solution (10µM) with STOP solution to a final concentration of 125nM. Make then a 1:2 serial dilution of 62.5nM, 31.25nM, 15.625nM, 7.8125nM, 3.90625nM, and 1.953125nM by adding 750µL to 750µL STOP solution and mix.

STOP buffer [0.2M glycine-NaOH, pH 10.4]: Dissolve 15g glycine (#36435.30, Alfa Aesar, USA) in 800ml MilliQ-water. Adjust pH with NaOH (#1.06498.1000, Merck KGaA, Germany) to 10.4 and fill up with MilliQ-water to a final volume of 1 liter. Filtrate through a 0.2µm filter (#28415-483, VWR International, USA).

Citrate/phosphate buffer [0.1M citric acid/0.2M Na_2_HPO_4_, pH 5]: Dissolve 19.2g citric acid (#84841.290, VWR Chemicals, USA) and 28.4g disodium phosphate (#28026.260, VWR Chemicals, USA) in 800ml MilliQ-water and adjust pH to 5. Fill up volume to 1 liter. Filtrate through a 0.2µm filter (#28415-483, VWR International, USA).

Assay buffer [Citrate/phosphate buffer, pH 5, 0.2% taurodeoxy-cholate]: Make about 20ml per 96-well plate, by adding 200mg TDC (#336840010, Acros organics, Belgium) per 100ml citrate/phosphate buffer (2mg/ml final concentration).

Substrate solution [0.5mM] Dissolve 4MU-β-glc (#J66630.MD, Sigma Aldrich, USA) in assay buffer to make a 0.5mM solution. Sonicate solution until completely dissolved. Filtrate through a 0.2µm filter (#28415-483, VWR International, USA).

DETAILED PROCEDURE

1. Thaw CSF samples on ice, mix and centrifuge (1000 x g for 1 min, 4°C).
2. Dilute samples with citrate/phosphate buffer.
3. Wash the 96-well plate (#3991, Corning, USA) twice with MilliQ water and let dry in the incubator.
4. Add 15µl of diluted sample to each well. Fill three wells per sample. Add 15µl citrate/phosphate buffer into the 24 spaces reserved for the calibrators.
5. With a multi-channel pipette, add 30µl substrate to each well.
6. Cover plate with sealing film (#732-4838, VWR International, USA) and shake plate for 3mins at 600rpm. Start the timer.
7. Incubate at 37±2°C for 3hrs in the dark.

START TIME: ___:___ END TIME ___:___

1. With a multi-channel pipette, add 180µl ice-cold STOP solution to each well except the 24 calibrator wells.
2. Add calibrator dilutions to the intended wells (180µL/well). Include triplicates. Add six replicates of the zero standard (= STOP solution).
3. Shake for 2mins at 600rpm. Read fluorescence at 446nm after excitation at 360nm.

INSTRUMENT SETTINGS (Biotek Synergy H1m, Gen5 Data Analysis software)

Read mode: Fluorescence intensity

Read position: Top

Excitation: 360nm

Emission: 446nm

Gain: 100

Read height: 7mm

INSTRUMENT SPECIFICATIONS (Biotek Synergy H1m)

Type: Multi-mode microplate reader

Optics: Double grating monochromators

Monochromator bandwidth (fluorescence): 16nm
